# Supplementary material for: The effect of support surface and footwear condition on postural sway and lower limb muscle action of the older women
Source: PLoS One. 2020 Jun 3;15(6):e0234140. doi: 10.1371/journal.pone.0234140 (PMC7269262; doi:10.1371/journal.pone.0234140)
Supplement: S1 Appendix — (DOCX) [file pone.0234140.s001.docx]

**S1 Appendix: Testing maneuver of maximum voluntary contractions**

The maximum voluntary contractions (MVC) of each muscle was acquired by manual resistance test. To evaluate the MVC of BF and VL, each participant was seated on a chair with a backrest placed against a wall, thighs strapped by a belt and feet placed on a stool with the knees flexed at 90°. Participants were asked to grasp the edge of the chair on each side for further stabilization. The tested lower leg was strapped using a non-elastic belt that was attached to a fixed structure and the participants were instructed to perform a maximal isometric knee extension (i.e. VL) or flexion (i.e. BF) by pulling against the belt for 5 seconds. To test the MVC during ankle plantarflexion (i.e., LG muscle) and dorsiflexion (i.e., TA muscle), the knee was placed in a fully extended position with support and the foot was placed in a neutral dorsiflexion/plantar-flexion position. To measure the MVC during ankle plantarflexion, the ankle was in a neutral position, and the participants were instructed to isometrically push the ankle against the wedge with maximal effort for 5 seconds. To test ankle dorsiflexion, the participants were asked to perform maximal isometric ankle dorsiflexion by pushing against the researcher’s hand for 5 seconds. Verbal encouragement in standard scripts was given during the contractions. Three trials were performed for each muscle, with a 5-minute rest interval between trials.
