# Supplementary material for: The effect of support surface and footwear condition on postural sway and lower limb muscle action of the older women
Source: PLoS One. 2020 Jun 3;15(6):e0234140. doi: 10.1371/journal.pone.0234140 (PMC7269262; doi:10.1371/journal.pone.0234140)
Supplement: S2 Appendix — (DOCX) [file pone.0234140.s002.docx]

**S2 Appendix: List of abbreviations**

| AP | Anterior-posterior |
| --- | --- |
| EMG | Electromyography |
| RMS | Root-mean-square |
| BF | Biceps femoris |
| C95 area | 95% confidence elliptical area |
| COP | Center of pressure |
| LG | Lateral gastrocnemius |
| ML | Medial-lateral |
| MVC | Maximum voluntary contractions |
| TA | Tibialis anterior |
| VL | Vastus lateralis |
