# Supplementary material for: The effect of support surface and footwear condition on postural sway and lower limb muscle action of the older women
Source: PLoS One. 2020 Jun 3;15(6):e0234140. doi: 10.1371/journal.pone.0234140 (PMC7269262; doi:10.1371/journal.pone.0234140)
Supplement: S1 File — (DOCX) [file pone.0234140.s006.docx]

**Laboratory Protocol**

**Inclusion Criteria**

**Participants can be included in this study if they:**

- Aged more than 60

AND

- Able to follow simple verbal instructions

AND

- No history of foot injuries during the past 2 years

**Anonymous participant IDs**

Each participant should be given an anonymous ID according to the following naming structure: *MELD_[site code]_number*

[site code] =  PW for laboratory at The Hong Kong Polytechnic University

[number] = 001, 002 etc.

Examples of participant IDs:

MELD_PW_001

MELD_PW_002

**Exclusion Criteria**

**Participants cannot be included in this study if:**

- have any neurological conditions and musculoskeletal problems that might affect balance

AND

- have contract or glaucoma

AND

- have impaired tactile foot sensitivity

**ID**

*For each participant include participant ID – named as specified above*

+

**Age**

*Age of participant in years at time of assessment for balance and postural control.*

+

**Procedures**

1. *Participants were interviewed to obtain their demographic information*
2. *Foot sensation test was conducted. Tactile foot sensitivity was evaluated using Semmes Weinstein Monofilament test (North Coast Medical Inc, California, United States)*
3. *Monofilaments, starting with 1.65 mm, were randomly applied to test regions including first, second, third toes, first, third, and fifth metatarsal heads, and medial and lateral arches of the midfoot.*
4. *Participants were asked to close eyes and indicate whether stimulations were perceived.*
5. *A maximum of three stimulations per monofilament was applied on each region.*
6. *The inability to detect 5.07 mm/10 g monofilament at first toes, first and third metatarsal heads was deemed as impaired tactile foot sensitivity*
7. *Participants who have impaired tactile foot sensitivity will be excluded for the following evaluation of balance and postural control at various footwear conditions.*
8. *Each participant underwent three different footwear conditions, namely barefoot, plain shoes made of soft terry textiles and nodulous insole shoes with medial arch support and silicone protrusions at the metatarsal heads and lateral heel in a randomized sequence*
9. *Participants were instructed to maintain quiet standing on firm (i.e., concrete floor) and foam surfaces (StimUp® Balance Pad) with their eyes open in the three footwear conditions, in a randomized order.*
10. *In standing posture, all the participants’ feet were 17 cm apart from the heel center, with the foot progression angle at 14°. They were asked to stand still with their hands by the sides and looking at a stationary visual target (i.e., a red spot of 2 cm in diameter) placed at eye level and 3-m in front of the participants and put equal body weight on each foot.*
11. *In each condition, participants were tested thrice for 30 seconds and a rest period of one minute was allowed between each condition. Data during the middle 20 seconds were used for analyses.*
12. *Static postural sway was measured as the displacement of center of pressure (COP) using in-shoe pressure measurement system* *(**Pedar®, Novel GmbH, Munich, Germany) with sampling frequency at 50 Hz under the dominant foot.*
13. *The COP parameters included the range of anterior-posterior (AP) and medial–lateral (ML) COP displacement,* *path length (PL) and the 95% confidence elliptical area (C95 area).*
14. *The surface EMG signal was captured using an 8-channel wireless EMG system (Clinical DTS,* *Noraxon USA Inc., AZ, USA).*
15. *After proper skin preparation, four circular Ag/AgCl bipolar electrodes were placed on the biceps femoris (BF), vastus lateralis (VL), tibialis anterior (TA), and lateral gastrocnemius (LG) of the dominant leg*
16. *Prior to postural stability test, maximum voluntary contractions (MVC) for each muscle was* *collected for 5 s using manual resistance and repeated three times with five minutes rest period in-between each MVC test.*
