# Supplementary material for: The effect of support surface and footwear condition on postural sway and lower limb muscle action of the older women
Source: PLoS One. 2020 Jun 3;15(6):e0234140. doi: 10.1371/journal.pone.0234140 (PMC7269262; doi:10.1371/journal.pone.0234140)
Supplement: Attachment — Submitted filename: comment review plos one.docx [file pone.0234140.s008.docx]

I am grateful for the possibility to revise this research study.

The effect of support surface and footwear condition on postural sway and lower limb

muscle action of the old people is a trend topic in the current research literature and may be a main focus of interest for readers.

Results of the abstract need to reflect the findings with respect to both groups and the lack of significant differences of balance, and also you need reflect the meaning of AP and ML because these abbreviates are not reflected clearly

Introduction may be improved adding new information in order to provide an adequate state-of-the-art including some references. I suggest to include this references include in the atteched to complet this requeriment

Lines 55-68

Rodríguez-Sanz D, Tovaruela-Carrión N, López-López D, Palomo-López P, Romero-Morales C, Navarro-Flores E, et al. Foot disorders in the elderly: A mini-review. Disease-a-Month. 2018 Mar;64(3):64–91.

Calvo-Lobo C, García AR, Iglesias MEL, López-López D, Rodríguez-Sanz D, Romero-Morales C, et al. The relationship between shoe fitting and foot health of persons with down syndrome: A case control study. Int J Environ Res Public Health. 2018 May 14;15(5).

López-López D, Marañon-Medina J, Losa-Iglesias ME, Calvo-Lobo C, Rodríguez-Sanz D, Palomo-López P, et al. The influence of heel height related on quality of life on the foot in a sample of women. Rev Assoc Med Bras. 2018 Apr;64(4):324–9.

Roca-Dols A, Elena Losa-Iglesias M, Sánchez-Gómez R, Becerro-de-Bengoa-Vallejo R, López-López D, Palomo-López P, et al. Electromyography activity of triceps surae and tibialis anterior muscles related to various sports shoes. J Mech Behav Biomed Mater. 2018/06/05. 2018 Oct;86:158–71.

Roca-Dols A, Losa-Iglesias ME, Sánchez-Gómez R, López-López D, Becerro-de-Bengoa-Vallejo R, Calvo-Lobo C. Electromyography comparison of the effects of various footwear in the activity patterns of the peroneus longus and brevis muscles. J Mech Behav Biomed Mater. 2018/03/13. 2018 Jun;82:126–32.

López-López D, Expósito-Casabella Y, Losa-Iglesias M, Bengoa-Vallejo RB de, Saleta-Canosa JL, Alonso-Tajes F. Impact of shoe size in a sample of elderly individuals. Rev Assoc Med Bras. 2016 Nov;62(8):789–94.

López López D, Losa Iglesias ME, Becerro de Bengoa Vallejo R, Palomo López P, Morales Ponce Á, Soriano Medrano A, et al. Optimal choice of footwear in the elderly population. Geriatr Nurs. 2015/08/12. 2015;36(6):458–61.

Roca-Dols A, Losa-Iglesias ME, Sánchez-Gómez R, Becerro-de-Bengoa-Vallejo R, López-López D, Rodríguez-Sanz D, et al. Effect of the cushioning running shoes in ground contact time of phases of gait. J Mech Behav Biomed Mater. 2018/08/25. 2018 Dec;88:196–200.

Methods are well-designed with relevant and complete information. Correct sample size calculations, good description of the properties of the outcome measurements as well as detailed statistical analyses were included. Tables, figures and redaction of the results are presented in a correct way providing a good presentation of the main finding of the study.

In line 76 to 82 in methods section I suggest authors must include a reference to Ethics requirements Helsinki declaration

Holt GR. Declaration of Helsinki—The World’s Document of Conscience and Responsibility. South Med J. 2014 Jul;107(7):407–407.

Discussion section may include future research studies secondary to the current findings of this study. Clinical considerations, limitations and overall discussion are well-presented, but future research may be useful in order to propose future research regarding this field.

Finally, I suggest to authors in discuss section to include the possbilty of influence of aging or even another pathologies like Parkinson diseease in your study finding suggest to include this references include in the attached to complete this requirement.

In line 198-214

Brognara, Navarro-Flores, Iachemet, Serra-Catalá, Cauli. Beneficial Effect of Foot Plantar Stimulation in Gait Parameters in Individuals with Parkinson’s Disease. Brain Sci. 2020 Jan 27;10(2):69.
