# Supplementary material for: The effect of support surface and footwear condition on postural sway and lower limb muscle action of the older women
Source: PLoS One. 2020 Jun 3;15(6):e0234140. doi: 10.1371/journal.pone.0234140 (PMC7269262; doi:10.1371/journal.pone.0234140)
Supplement: Attachment — Submitted filename: Response to Reviewers.docx [file pone.0234140.s010.docx]

**Response to Reviewers**

**First of all, we would like to thank the academic editor and reviewers for their constructive comments, which are very helpful in improving the quality of the manuscript. We have addressed all the comments raised in the revised manuscript. The point-by-point responses to the comments are provided below.**

**Academic editor:**

To enhance the reproducibility of your results, we recommend that if applicable you deposit your laboratory protocols in protocols.io, where a protocol can be assigned its own identifier (DOI) such that it can be cited independently in the future.

**Response: Thank you for your recommendation. We have added laboratory protocols following the guidelines.**

**Reviewer #1:**

1. There is repeated information on ethics approval on line 90 and 99. Please revise accordingly.
   **Response: Thank you for the error. We have removed the repeated information as suggested.**
2. 23 participants involved in this study (line 180) but this number is not tally with the reported number of the dominant leg (line 184). Please revise accordingly.
   **Response: Rectification has been made accordingly (Line 182).**
